# Supplementary material for: COZOID: contact zone identifier for visual analysis of protein-protein interactions
Source: BMC Bioinformatics. 2018 Apr 6;19:125. doi: 10.1186/s12859-018-2113-6 (PMC5889581; doi:10.1186/s12859-018-2113-6)
Supplement: Supplementary file 2 — Software build. Executable binary file of the software tool. (ZIP 118,784 kb) [file 12859_2018_2113_MOESM2_ESM.zip › cozoid/platform/docs/testng-6.5.1-javadoc.zip!/org/testng/TimeBombSkipException.html]

TimeBombSkipException (TestNG)


---


|  |  |  |
| --- | --- | --- |
| **Overview** \| **Package** \| **Class** \| **Tree** \| **Deprecated** \| **Index** \| **Help** | |  |
| **PREV CLASS**   NEXT CLASS | **FRAMES**    **NO FRAMES**     **All Classes** |  |
| SUMMARY: NESTED \| FIELD \| CONSTR \| METHOD | DETAIL: FIELD \| CONSTR \| METHOD |  |


---


## org.testng Class TimeBombSkipException

```
java.lang.Object
  java.lang.Throwable
      java.lang.Exception
          java.lang.RuntimeException
              org.testng.SkipException
                  org.testng.TimeBombSkipException
```

**All Implemented Interfaces:**: java.io.Serializable

---

``` public class TimeBombSkipException extends SkipException ```

A `SkipException` extension that transforms a skipped method
into a failed method based on a time trigger.

By default the time format is yyyy/MM/dd (according to `SimpleDateFormat`).
You can customize this by using the specialized constructors. Suppported date
formats are according to the `SimpleDateFormat`.

**Since:**
:   5.6

**Author:**
:   Alex Popescu

**See Also:**: Serialized Form

---

**Constructor Summary**

`TimeBombSkipException(java.lang.String msg,
java.util.Date expirationDate)`
  
          Creates a `TimeBombedSkipException` using the expirationDate.

`TimeBombSkipException(java.lang.String msg,
java.util.Date expirationDate,
java.lang.String format)`
  
          Creates a `TimeBombedSkipException` using the expirationDate.

`TimeBombSkipException(java.lang.String msg,
java.util.Date expirationDate,
java.lang.String format,
java.lang.Throwable cause)`
  
          Creates a `TimeBombedSkipException` using the expirationDate.

`TimeBombSkipException(java.lang.String msg,
java.util.Date expirationDate,
java.lang.Throwable cause)`
  
          Creates a `TimeBombedSkipException` using the expirationDate.

`TimeBombSkipException(java.lang.String msg,
java.lang.String date)`
  
          Creates a `TimeBombedSkipException` using the date
in the format yyyy/MM/dd.

`TimeBombSkipException(java.lang.String msg,
java.lang.String date,
java.lang.String format)`
  
          Creates a `TimeBombedSkipException` using the date
in the specified format format.

`TimeBombSkipException(java.lang.String msg,
java.lang.String date,
java.lang.String inFormat,
java.lang.String outFormat)`
  
          Creates a `TimeBombedSkipException` using the date
in the specified format inFormat.

`TimeBombSkipException(java.lang.String msg,
java.lang.String date,
java.lang.String inFormat,
java.lang.String outFormat,
java.lang.Throwable cause)`
  
          Creates a `TimeBombedSkipException` using the date
in the specified format inFormat.

`TimeBombSkipException(java.lang.String msg,
java.lang.String date,
java.lang.String format,
java.lang.Throwable cause)`
  
          Creates a `TimeBombedSkipException` using the date
in the specified format format.

`TimeBombSkipException(java.lang.String msg,
java.lang.String date,
java.lang.Throwable cause)`
  
          Creates a `TimeBombedSkipException` using the date
in the format yyyy/MM/dd.


| **Method Summary** | |
| --- | --- |
| `java.lang.String` | `getMessage()` |
| `boolean` | `isSkip()`             Flag if the current exception marks a skipped method (true) or a failure (false). |
| `void` | `printStackTrace(java.io.PrintStream s)` |
| `void` | `printStackTrace(java.io.PrintWriter s)` |

**Methods inherited from class org.testng.SkipException**

`reduceStackTrace, restoreStackTrace`

**Methods inherited from class java.lang.Throwable**

`fillInStackTrace, getCause, getLocalizedMessage, getStackTrace, initCause, printStackTrace, setStackTrace, toString`

**Methods inherited from class java.lang.Object**

`clone, equals, finalize, getClass, hashCode, notify, notifyAll, wait, wait, wait`

**Constructor Detail**

### TimeBombSkipException

```
public TimeBombSkipException(java.lang.String msg,
                             java.util.Date expirationDate)
```

:   Creates a `TimeBombedSkipException` using the expirationDate.
    The format used for date comparison is yyyy/MM/dd

    **Parameters:**: `msg` - exception message: `expirationDate` - time limit after which the SKIP becomes a FAILURE

---


### TimeBombSkipException

```
public TimeBombSkipException(java.lang.String msg,
                             java.util.Date expirationDate,
                             java.lang.String format)
```

:   Creates a `TimeBombedSkipException` using the expirationDate.
    The format parameter wiil be used for performing the time comparison.

    **Parameters:**: `msg` - exception message: `expirationDate` - time limit after which the SKIP becomes a FAILURE: `format` - format for the time comparison

---


### TimeBombSkipException

```
public TimeBombSkipException(java.lang.String msg,
                             java.lang.String date)
```

:   Creates a `TimeBombedSkipException` using the date
    in the format yyyy/MM/dd.

    **Parameters:**: `msg` - exception message: `date` - time limit after which the SKIP becomes a FAILURE

---


### TimeBombSkipException

```
public TimeBombSkipException(java.lang.String msg,
                             java.lang.String date,
                             java.lang.String format)
```

:   Creates a `TimeBombedSkipException` using the date
    in the specified format format. The same format is used
    when performing the time comparison.

    **Parameters:**: `msg` - exception message: `date` - time limit after which the SKIP becomes a FAILURE: `format` - format of the passed in date and of the time comparison

---


### TimeBombSkipException

```
public TimeBombSkipException(java.lang.String msg,
                             java.lang.String date,
                             java.lang.String inFormat,
                             java.lang.String outFormat)
```

:   Creates a `TimeBombedSkipException` using the date
    in the specified format inFormat. The outFormat will be
    used to perform the time comparison and display.

    **Parameters:**: `msg` - exception message: `date` - time limit after which the SKIP becomes a FAILURE: `inFormat` - format of the passed in date: `outFormat` - format of the time comparison

---


### TimeBombSkipException

```
public TimeBombSkipException(java.lang.String msg,
                             java.util.Date expirationDate,
                             java.lang.Throwable cause)
```

:   Creates a `TimeBombedSkipException` using the expirationDate.
    The format used for date comparison is yyyy/MM/dd

    **Parameters:**: `msg` - exception message: `expirationDate` - time limit after which the SKIP becomes a FAILURE: `cause` - the cause (which is saved for later retrieval by the `Throwable.getCause()` method). (A null value is permitted, and indicates that the cause is nonexistent or unknown.)

---


### TimeBombSkipException

```
public TimeBombSkipException(java.lang.String msg,
                             java.util.Date expirationDate,
                             java.lang.String format,
                             java.lang.Throwable cause)
```

:   Creates a `TimeBombedSkipException` using the expirationDate.
    The format parameter wiil be used for performing the time comparison.

    **Parameters:**: `msg` - exception message: `expirationDate` - time limit after which the SKIP becomes a FAILURE: `format` - format for the time comparison: `cause` - the cause (which is saved for later retrieval by the `Throwable.getCause()` method). (A null value is permitted, and indicates that the cause is nonexistent or unknown.)

---


### TimeBombSkipException

```
public TimeBombSkipException(java.lang.String msg,
                             java.lang.String date,
                             java.lang.Throwable cause)
```

:   Creates a `TimeBombedSkipException` using the date
    in the format yyyy/MM/dd.

    **Parameters:**: `msg` - exception message: `date` - time limit after which the SKIP becomes a FAILURE: `cause` - the cause (which is saved for later retrieval by the `Throwable.getCause()` method). (A null value is permitted, and indicates that the cause is nonexistent or unknown.)

---


### TimeBombSkipException

```
public TimeBombSkipException(java.lang.String msg,
                             java.lang.String date,
                             java.lang.String format,
                             java.lang.Throwable cause)
```

:   Creates a `TimeBombedSkipException` using the date
    in the specified format format. The same format is used
    when performing the time comparison.

    **Parameters:**: `msg` - exception message: `date` - time limit after which the SKIP becomes a FAILURE: `format` - format of the passed in date and of the time comparison: `cause` - the cause (which is saved for later retrieval by the `Throwable.getCause()` method). (A null value is permitted, and indicates that the cause is nonexistent or unknown.)

---


### TimeBombSkipException

```
public TimeBombSkipException(java.lang.String msg,
                             java.lang.String date,
                             java.lang.String inFormat,
                             java.lang.String outFormat,
                             java.lang.Throwable cause)
```

:   Creates a `TimeBombedSkipException` using the date
    in the specified format inFormat. The outFormat will be
    used to perform the time comparison and display.

    **Parameters:**: `msg` - exception message: `date` - time limit after which the SKIP becomes a FAILURE: `inFormat` - format of the passed in date: `outFormat` - format of the time comparison: `cause` - the cause (which is saved for later retrieval by the `Throwable.getCause()` method). (A null value is permitted, and indicates that the cause is nonexistent or unknown.)


**Method Detail**

### isSkip

```
public boolean isSkip()
```

:   **Description copied from class: `SkipException`**
:   Flag if the current exception marks a skipped method (true)
    or a failure (false). By default Subclasses should override this method
    in order to provide smarter behavior.

    :   **Overrides:**: `isSkip` in class `SkipException`
    :   **Returns:**: true if the method should be considered a skip, false if the method should be considered failed. If not overwritten it returns true

---


### getMessage

```
public java.lang.String getMessage()
```

:   **Overrides:**: `getMessage` in class `java.lang.Throwable`

---


### printStackTrace

```
public void printStackTrace(java.io.PrintStream s)
```

:   **Overrides:**: `printStackTrace` in class `java.lang.Throwable`

---


### printStackTrace

```
public void printStackTrace(java.io.PrintWriter s)
```

:   **Overrides:**: `printStackTrace` in class `java.lang.Throwable`


---


|  |  |  |
| --- | --- | --- |
| **Overview** \| **Package** \| **Class** \| **Tree** \| **Deprecated** \| **Index** \| **Help** | |  |
| **PREV CLASS**   NEXT CLASS | **FRAMES**    **NO FRAMES**     **All Classes** |  |
| SUMMARY: NESTED \| FIELD \| CONSTR \| METHOD | DETAIL: FIELD \| CONSTR \| METHOD |  |


---
